# Arabic-Translated Versions of Patient-Reported Outcome Measures Utilized in Spine Research: A Review of Validated Studies

**DOI:** 10.7759/cureus.46303

**Published:** 2023-10-01

**Authors:** Abdulhakim B Jamjoom, Abdulhadi Y Gahtani, Moajeb T Alzahrani, Laila M Baydhi, Ahmad S Albeshri, Momen Sharab

**Affiliations:** 1 Neurosurgery, King Saud Bin Abdulaziz University for Health Sciences College of Medicine, Jeddah, SAU; 2 Neurosurgery, King Abdulaziz Medical City Western Region, Jeddah, SAU

**Keywords:** surveys and questionnaires, reliability, validity, measurement properties, cross-cultural adaptation, translation, arabic, proms, patient-reported outcome measures

## Abstract

Patient-reported outcome measures (PROMs) are standardized tools commonly applied in research and healthcare appraisal. Most were developed in English and the cross-cultural adaptation (CCA) and validation of their translated versions remain topics of contemporary research appeal. This review aimed to identify the Arabic-translated PROMs that were utilized in spine research and to assess the methodological qualities of their studies. The PubMed database was searched, and all relevant publications were identified. The CCA and measurement properties were assessed using the guidelines described by Oliveria and Terwee respectively. Thirty studies that validated the Arabic versions of 26 PROMs were found suitable. The tools that had the highest total citation numbers were Neck Disability Index, Ronald-Morris Disability Questionnaire, Oswestry Disability Index, Fear Avoidance Beliefs Questionnaire, Scoliosis Research Society-22, Back Beliefs Questionnaire, Quebec Back Pain Disability Scale, and McGill Pain Questionnaire-Short Form. The Arabic versions of Short Form-36 (SF-36), Visual Analogue Scale (VAS), and EuroQol-5D (EQ-5D) were not included due to lack of validation in spine research. All the articles were published from 2007 to 2023 (median 2019) and their journal’s impact factor and citation numbers were relatively modest (mean 2 and 6.5 respectively). Most patients had low back pain (19 articles), were recruited from physiotherapy and rehabilitation departments (18 articles) and came from the Kingdom of Saudi Arabia (12 articles). The quality of the CCA of the Arabic versions was rated good in forward translation, synthesis, back translation, and expert committee review but less so in pretesting and submission. The measurement properties of the studies were considered good quality in internal consistency, reliability, structural validity and cross-cultural validity but less so in content validity, error measurement, responsiveness and floor/ceiling effect. In conclusion, with a few exceptions, most of the widely utilized PROMs in spine research have validated Arabic versions. The methodological quality of the studies was good apart from a few shortages that could be improved upon by further research. Work should be done to address the validation of Arabic versions of SF-36, VAS and EQ-5D in spine research. PROMs are valuable in systematizing subjective outcomes. Their usage in research and clinical settings in any validated language should be highly encouraged.

## Introduction and background

Patient-reported outcome measures (PROMs) are validated questionnaires that assess patients’ health status and are completed by the patients themselves without interpretation by clinicians or anyone else. The term covers an extensive range of instruments that measure quality of life, well-being, satisfaction, symptoms, and functioning [1-3]. PROMs are widely accepted as valuable tools in research, clinical decision-making, patient-centred care, health policy and more recently compensation rulings. They are designed to be generic, disease-specific, or treatment-specific [4-6]. For the tools to be useful, they must possess several quality properties and pass stringent methodological appraisals that include validity, reliability, responsiveness, and interpretability [6,7]. The utilization of PROMs in spine research is well documented. The nature and number of instruments used vary according to the breadth of the search criteria. Guzman et al. [5], identified 206 tools that were used in studies that were published in five spine journals from 2004 to 2013. Beighley et al. [8] named 37 spine-specific PROMs that were utilized the spine disease and deformity literature. Ramasamy et al. [9] found 176 PROMs that were employed in chronic back pain research during 2011 and 2015. Jamjoom et al. [4] reported 33 PROMs that were reported in high-impact publications in the neurospine surgical literature. Most PROMs were developed in the English language and the literature is rich with research initiatives that focused on translating them to other languages. It is accepted that for a translated version to be acknowledged as having similar properties to the original tool it must meet rigorous assessments of its cross-cultural adaptation (CCA) and validation [1-3]. This matter has been a topic of significant research interest in recent years. In fact, 19 out of the 50 most cited publications that utilized PROMs in the neurospine surgical literature focused on validating translated versions of PROMs to other languages [4].

Arabic is one of the most commonly spoken languages in the world. It is the official language in 25 countries and is articulated by more than 300 million people [1,10]. Arab countries’ contribution to the neurospine surgical literature may be judged modest. Their researchers published 434 articles in the spine literature over a 15-year period (2000-2015) [11] and accounted for only 0.53% of the total neurosurgery research from 2005 to 2019 [12]. Most Arab countries are considered developing countries and many of them are counted as low- and middle-income on the economic scale [12]. Lack of resources, absence of research infrastructure and deficiency of state-of-the-art technology were identified as the reasons why the Arab world lagged behind in biomedical research [12]. Nevertheless, an increase in academic productivity in Arab countries has been observed in the spine [11,13], and neurosurgical research in recent years [12]. The Arabic language has many different dialects, however, modern standard Arabic is widely understood and accepted in formal communication and the media in all Arab countries. In the last two years, three studies reviewed the quality of the Arabic translations of PROMs that were utilized in a wide range of diseases [1,2] or related to a specific anatomical location [10]. To our knowledge, an evaluation of the Arabic versions of spine-related PROMs has not been addressed in the literature. This review aimed at identifying and assessing the methodological quality of the Arabic-translated, cross-culturally adapted, and validated PROMs that were utilized in spine research.

## Review

Methods

This study was carried out at King Saud bin Abdulaziz University for Health Science (KSAU-HS), Jeddah, Kingdom of Saudi Arabia (KSA). No ethical approval was necessary by our institution as the study was based on data obtained from open-access sources. The PubMed database was searched in June 2023 for studies that validated Arabic translations of PROMs utilized in spine literature. The search was performed using the keywords alone or in combination: “Arabic,” “Arab,” “Spine,” “Spinal,” “Scoliosis,” “Back,” “Neck,” “Validation,” “Validity,” “Reliability,” “Cross-Cultural,” “Cervical,” “Lumbar, and “Dorsal.” Using full articles, the following data was collected for each study: year of publication, publishing journal, its impact factor (IF), number of authors, number of centres, citation number, PROM name and number of items, number of participating patients, their country, the diagnoses for which the PROMs were used and the setting where the recruitment was done. The journal IF data was obtained from an online source [14]. The statistical analysis was carried out using Social Sciences Statistics [15]. In view of the regular changes in the citation numbers, the search findings on a single day (1st August 2023) were documented and used for analysis.

The CCA was assessed using the guidelines described by Oliveria et al. [2,16,17]. The guidelines comprise the following six items: initial translation, synthesis, back translation, expert committee review, pretesting and submission. For every study, each of the items was rated as good, fair, or not assessed. A positive rating based on the Oliveria et al. criteria [2,16,17] was given a good rating while doubtful and negative ratings were allocated a fair rating. The measurement properties were evaluated using the guidelines described by Terwee et al. [2,17,18]. The parameters consisted of the following eight items: internal consistency, reliability, structural validity, content validity, cross-cultural validity, error measurement, responsiveness, and floor/ceiling effect. For every study, each of the items was rated as good, fair, or not assessed. A positive rating based on the Terwee et al. criteria [2,17,18] was given a good rating while doubtful and negative ratings were allocated a fair rating. The collection of data, CCA and measurement properties assessments were performed by two of the authors independently and any discrepancies were resolved by consensus.

Results

**Figure 1:**
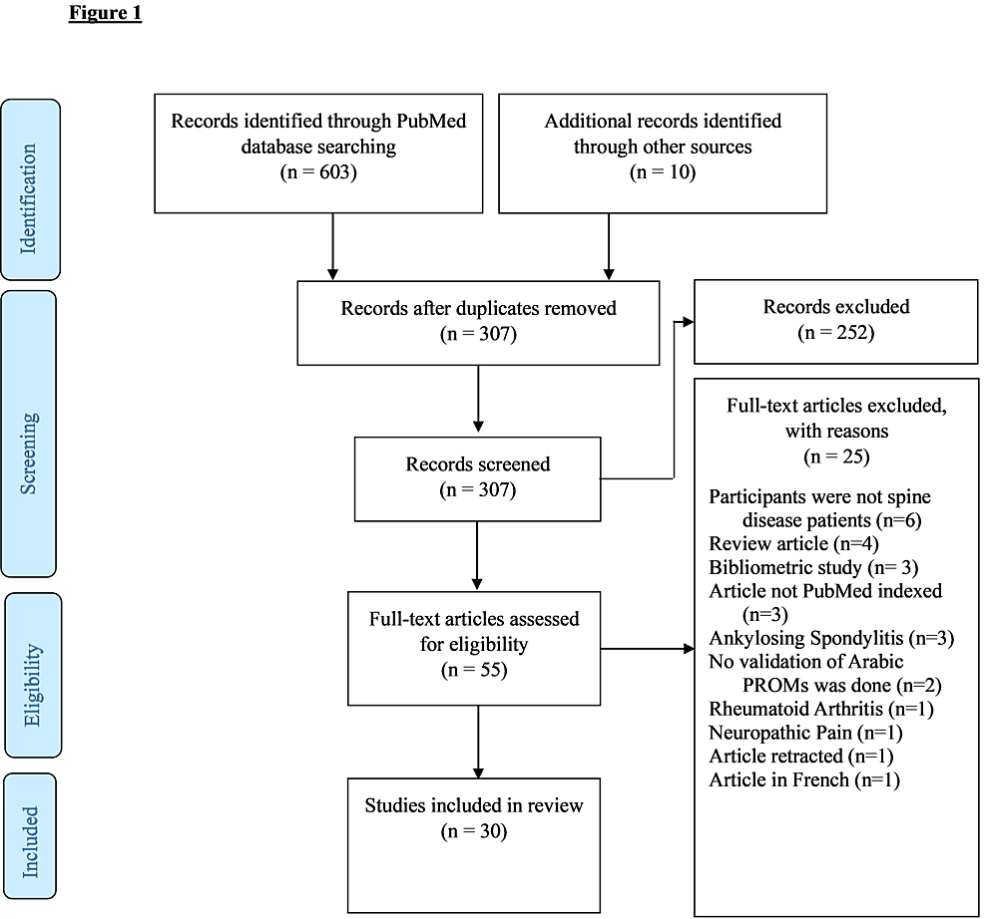
Preferred Reporting Items for Systematic Reviews and Meta-Analyses (PRISMA) flowchart for the review of Arabic-translated versions of patient-reported outcome measures (PROMs) utilized in spine research

The search yielded a total of 613 articles. After removing duplicates, title and abstract screening, and full-text review, 583 articles were excluded. A Preferred Reporting Items for Systematic Reviews and Meta-Analyses (PRISMA) flow diagram showing the flow of the review phases is presented in Figure 1.

The 30 articles that satisfied the inclusion criteria are summarized in Table 1 [19-48]. Four studies reported PROMs that had already been translated into Arabic [24,27,28,39]. Hence, the selected studies provided Arabic versions of 26 tools. Of the latter, 24 were considered disease-specific while two (Patient Health Questionnaire-9 [33], and Pain Self-Efficacy Questionnaire [48]) were judged generic.

**Table 1:** Analysis of the selected studies of Arabic-translated versions of patient-reported outcome measures (PROMs) utilized in the spine surgery literature ranked in chronological order. RMDQ: Roland-Morris Disability Questionnaire, FABQ: Fear Avoidance Beliefs Questionnaire, QBPDS: Quebec Back Pain Disability Scale, NDI: Neck Disability Index, ODI: Oswestry Disability Index, SRS-22: Scoliosis Research Society Questionnaires-22, BBQ: Back Beliefs Questionnaire, JOA-BPEQ: Japanese Orthopaedic Association Back Pain Evaluation Questionnaire, MPQ-SF: McGill Pain Questionnaire Short Form, CNFDS: Copenhagen Neck Functional Disability Scale, SBT: STarT Back Tool, PHQ 9: Patient Health Questionnaire-9, EOS-24: Early Onset Scoliosis 24 Items Questionnaire, COMI: Core Outcome Measures Index, BQ: Bournemouth questionnaire, TSK: Tampa Scale of Kinesiophobia, mJOA-CMS: Modified Japanese Orthopaedic Association Cervical myelopathy score, ISYQOL: Italian Spine Youth Quality of Life, Back-PAQ: Back Pain Attitudes Questionnaire, LBP-KQ: Low Back Pain Knowledge Questionnaire, LBP-TBQ: Low Back Pain Treatment Beliefs Questionnaire, FRI: Functional Rating Index, SRS-30: Scoliosis Research Society Questionnaires-30, OMPQ: Orebo Musculoskeletal Pain Questionnaire, NBSS-SF: Neurogenic Bladder Symptoms Score-Short Form, PSEQ: Pain Self-Efficacy Questionnaire, LBP: low back pain, CSM: cervical spondylotic myelopathy, KSA: Kingdom of Saudi Arabia, Partic.: Participants, No.: number

First Author	Journal	Year	PROMs Name	Partic. No.	Partic. Setting	Partic. Country	Cites
Maaroufi H [19]	Spine	2007	RMDQ	76	Rheumatology	Morocco	45
Laufera Y [20]	J Back Musculo Rehabil	2012	FABQ	122	Physiotherapy	Arab Israel	24
Alnahhal A [21]	Spine	2012	QBPDS	148	Physiotherapy	Palestine	31
Shaheen A [22]	Spine	2013	NDI	65	Primary Care	KSA	97
Algarni A [23]	Ann Physical Rehab Med	2014	ODI	100	Physiotherapy	KSA	70
Maki D [24]	Spine	2014	RMDQ	201	Physiotherapy	Bahrain	34
Haidar R [25]	Spine	2015	SRS-22	81	Orthopedics	Lebanon	38
Alamrani S [26]	Spine	2016	BBQ	115	Physiotherapy	KSA	19
Alanazi F [27]	Spine	2017	FABQ	70	Physiotherapy	KSA	30
Maki D [28]	Disabil Rehabil	2017	BBQ	199	Physiotherapy	Bahrain	14
Alfayez S [29]	J Ortho Sci	2017	JOA-BPEQ	151	Orthopedics	KSA	13
Terkawi A [30]	Saudi J Anaesth	2017	MPQ-SF	142	Pain Clinic	KSA	26
Elbeltagy A [31]	Asian Spine Journal	2018	CNFDS	74	Physiotherapy	Egypt	5
Elsabbagh L [32]	J Ortho Sci	2018	SBT	59	Physiotherapy	KSA	10
Summaka M [33]	World Neurosurgery	2019	PHQ-9	51	Physiotherapy	Lebanon	7
Hanbali Y [34]	SICOT-J	2019	EOS-24	58	Orthopedics	Palestine	14
Abdeldaiem A [35]	Europ Spine J	2020	COMI	85	Physiotherapy	Egypt	5
Elerian A [36]	Physiother Res Int.	2020	BQ	70	Physiotherapy	Egypt	3
AL-Shudifat A [37]	Medicine (Baltimore)	2020	TSK	101	Neurosurgery	Jordan	6
Fawaz S [38]	Spine Surg Relat Res	2020	mJOA-CMS	65	Spine	Egypt	0
Elnady B [39]	SICOT-J	2021	mJOA-CMS	100	Spine	Egypt	3
Fallatah S [40]	Medicine (Baltimore)	2021	ISYQOL	115	Spine	KSA	2
Kanaan S [41]	J Back Musculo Rehab	2021	Back-PAQ	110	Physiotherapy	Jordan	5
Kanaan S [42]	Physiother Theory Pract	2022	LBP-KQ	124	Physiotherapy	Jordan	0
Kanaan S [43]	J Back Musculo Rehabil	2023	LBP-TBQ	51	Physiotherapy	Jordan	4
Alsaadi S [44]	Rehabil Res Practice	2022	FRI	200	Physiotherapy	KSA	0
Alzakri A [45]	Spine Deform	2023	SRS-30	322	Orthopedics	KSA	0
Alanazi F [46]	J Pain Research	2023	OMPQ	84	Primary Care	KSA	0
Khadour Y [47]	J Orthop Surg Res	2023	NBSS-SF	101	Physiotherapy	Syria	0
Almutairi B [48]	Physiother Theory Pract	2023	PSEQ	113	Physiotherapy	KSA	3

A full list of the 26 PROMs is shown in Table 2. The median (range) number of items per PROM was 16 (7-30). The median (range) article citation number was 6.5 (0-97). The tools with the highest total citations numbers were Neck Disability Index (NDI) (97) [22], Ronald-Morris Disability Questionnaire (RMDQ) (79) [19,24], Oswestry Disability Index (ODI) (70) [23], Fear Avoidance Beliefs Questionnaire (FABQ) (54) [20,27], Scoliosis Research Society-22 (SRS-22) (38) [25], Back Beliefs Questionnaire (BBQ) (33) [26,28], Quebec Back Pain Disability Scale (QBPDS) (31) [21], and McGill Pain Questionnaire-Short Form (MPQ-SF) (26) [30].

**Table 2:** List of the 26 validated cross-culturally adapted translated Arabic versions of patient-reported outcome measures (PROMs) that were utilized in spine literature.

Disease	PROMs Name	PROMs Abbreviation
Low Back Pain	Roland-Morris Disability Questionnaire	RMDQ [19,24]
	Fear Avoidance Beliefs Questionnaire	FABQ [20,27]
	Back Beliefs Questionnaire	BBQ [26,28]
	Quebec Back Pain Disability Scale	QBPDS [21]
	Oswestry Disability Index	ODI [23]
	Japanese Orthopaedic Association Back Pain Evaluation Questionnaire	JOA-BPEQ [29]
	STarT Back Tool	SBT [32]
	Core Outcome Measures Index	COMI [35]
	Bournemouth questionnaire	BQ [36]
	Tampa Scale of Kinesiophobia	TSK [37]
	Back Pain Attitudes Questionnaire	Back-PAQ [41]
	Low Back Pain Knowledge Questionnaire	LBP-KQ [42]
	Low Back Pain Treatment Beliefs Questionnaire	LBP-TBQ [43]
	Functional Rating Index	FRI [44]
	Orebo Musculoskeletal Pain Questionnaire	OMPQ [46]
	Pain Self-Efficacy Questionnaire	PSEQ [48]
Neck Pain and Cervical Myelopathy	Modified Japanese Orthopaedic Association Cervical Myelopathy Score	mJOA-CMS [38,39]
	Neck Disability Index	NDI [22]
	Copenhagen Neck Functional Disability Scale	CNFDS [31]
Spine Pain	McGill Pain Questionnaire-Short Form	MPQ-SF [30]
Scoliosis	Scoliosis Research Society Questionnaire-22	SRS-22 [25]
	Early Onset Scoliosis 24 Items Questionnaire	EOS-24 [34]
	Italian Spine Youth Quality of Life	ISYQOL [40]
	Scoliosis Research Society Questionnaire-30	SRS-30 [45]
Spinal Cord Injury	Patient Health Questionnaire-9	PHQ-9 [33]
	Neurogenic Bladder Symptoms Score-Short Form	NBSS-SF [47]

The median (range) article publication year and age were 2019 (2007-2023) and four (0.5-17) years respectively. The median (range) journal IF was 2 (0.2 to 5.4). The median (range) number of authors, centres and countries per article were five (1-9), three (1-7) and two (1-6) respectively. The most common publishing journal was Spine (Phila Pa 1976) (seven articles). The journals’ specialties were spine (11 articles), physiotherapy and rehabilitation (nine articles), orthopaedics (five articles), neurosurgery (one article) and others (four articles). The median (range) number of participants was 101 (51-322). The participants’ countries were KSA (12 articles), Egypt (five articles), Jordan (four articles), Lebanon (two articles), Bahrain (two articles), Palestine (two articles), and single articles from each of Israel, Syria and Morocco. The participants’ diseases were low back pain (LBP) (19 articles), neck pain and cervical spondylotic myelopathy (four articles), scoliosis (four articles), spinal cord injury (SCI) (two articles) and mixed spinal pain (one article). The participants’ settings were the services of physiotherapy and rehabilitation medicine (18 articles), spine, orthopaedics and neurosurgery (eight articles) and others (four articles).

The results of the quality assessment of the CCA are summarized in Table 3. Most studies had good ratings in initial translation (83%), synthesis (77%), back translation (87%) and expert committee review (83%). However, the good rating score was lower in pretesting (23%) and submission (47%).

**Table 3:** Summary of the cross-cultural adaptation (CCA) overall assessment for the reviewed 30 articles according to the guidelines described by Oliveria et al. *Good: The CCA rating meets Oliveria et al. [2,16,17] assessment for a positive rating **Fair: The CCA rating meets Oliveria et al. [2,16,17] assessment for doubtful and negative ratings

CCA Parameter	*Good rating (%)	**Fair rating (%)	Not assessed (%)
Initial Translation	25(83%) [19,20,22,24-28,30-32,34-36,38-48]	2(7%) [23,29]	3(10%) [21,33,37]
Synthesis	23(77%) [19,22,24,26-28,30-32,34-36,38-48]	2(7%) [20,25]	5(17%) [21,23,29,33,37]
Back Translation	26(87%) [19,20,22,24-32,34-36,38-48]	1(3%) [23]	3(10%) [21,33,37]
Expert Committee Review	25(83%) [19,20,22-28,30-32,34-36,39-48]	0	5(17%) [21,29,33,37,38]
Pretesting	7(23%) [23,26,27,32,35,44,45]	13(43%) [19,20,22,24,29,39-43,46-48]	10(33%) [21,25,28,30,31,33,34,36-38]
Submission	14(47%) [19,22,26,28,32,35,36,40-44,46,48]	0	16(53%) [20,21,23-25,27,29-31,33,34,37-39,45,47]

The findings related to the quality of the measurement properties are demonstrated in Table 4. Most articles received good ratings for internal consistency (87%), reliability (73%), structural validity (63%) and cross-cultural validity (53%). However, the good rating score was lower for content validity (30%), error measurement (13%), responsiveness (7%) and floor/ceiling effect (43%).

**Table 4:** Summary of the measurement properties overall assessment for the reviewed 30 articles according to the guidelines described by Terwee et al. *Good: The measurement property rating meets Terwee et al. [2,17,18] assessment for a positive rating **Fair: The measurement property rating meets Terwee et al. [2,17,18] assessment for doubtful and negative ratings

Measurement Properties Parameter	*Good rating (%)	**Fair rating (%)	Not assessed (%)
Internal consistency	26(87%) [19-26,28-34,36-42,44,45,47,48]	1(3%) [43]	3(10%) [27,35,46]
Reliability	22(73%) [19-28,30,33,36,38,39,41-45,47,48]	2(7%) [35,46]	6(20%) [29,31,32,34,37,40]
Structural validity	19(63%) [19,21-23,26,28,30,32,35,36,39-44,46-48]	4(13%) [20,24,27,33]	7(23%) [25,29,31,34,37,38,45]
Content validity	9(30%) [21,23,25,32,36,41,42,46,47]	2(7%) [31,33]	19(63%) [19,20,22,24,26-30,34,35,37-40,43-45,48]
Cross-cultural validity	16(53%) [19,20,22,26,27,32-35,41-44,46-48]	3(10%) [25,39,40]	11(37%) [21,23,24,28-31,36-38,45]
Error measurement	4(13%) [25,35,44,48]	5(17%) [24,26,39,41,43]	21(70%) [19-23,27-34,36-38,40,42,45-47]
Responsiveness	2(7%) [22,27]	4(13%) [30,31,33,43]	24(80%) [19-21,23-26,28,29,32,34-42,44-48]
Floor/ceiling effect	13(43%) [20,22,25,26,32,34-36,39,41,45,46,48]	2(7%) [40,42]	15(50%) [19,21,23,24,27-31,33,37,38,43,44,47]

Discussion

PROMs aid surgeons and therapists in assessing patients’ outcomes in systematized ways that are tailored to the patients’ management and in a manner that allows them to engage with their healthcare providers. Translated, cross-culturally adapted and validated versions of PROMs are increasingly utilized in spine research in Arab countries [19-48]. Matters such as understanding, experiences, religious beliefs, and expectations of patients and primary healthcare practitioners have also been looked into in recent publications [49,50]. None of the tools identified here was included amongst the 26 instruments listed in the recent systematic review that reported an assortment of PROMs in Arab-speaking populations [1]. Furthermore, the newly published scoping review of 317 validated Arabic PROMs [2], included only nine of the 26 tools in this review [19,21-24,27,32,34,36]. Some of the tools reviewed here are recognized to be amongst the most commonly used in spine research in the literature. These include RMDQ [4-6,8,9], ODI [4-6,8,9], FABQ [4,5], BBQ [5], SRS-22 [4,5,8], NDI [4,5,8], QBPDS [5,6,8], MPQ-SF [5,9], Japanese Orthopaedic Association (JOA) Scale [4,5,8], Tampa Scale of Kinesiophobia (TSK) [4,5], Core Outcome Measure Index (COMI) [5,8], Orebo Musculoskeletal Pain Questionnaire [8] and the Bournemouth Questionnaire [8]. A notable absence from the list of 26 PROMs shown in Table 2 were four tools that are used in spine research. These are Short Form-36 (SF-36) [4,5], Visual Analogue Scale (VAS) [4,5], EuroQol-5D (EQ-5D) [5], and Zurich Claudication Questionnaire (ZCQ) [5,8]. Three of these instruments (SF-36 [1,2,51], EQ-5D [3,52] and NRS [53]), had Arabic-translated versions but they were not included in this review because their validation was in random population and in not spine research. VAS was used to assess Arab patients in one study that did not address validation [49], while to our knowledge, ZCQ has not been translated and validated in Arabic.

Fourteen (47%) of the articles reviewed here were published during 2020 to 2023 [35-48], an indication that validation of Arabic versions of spine-related PROMs is an ongoing topic in the literature. Article citation numbers are known to positively correlate with journal IF and publication's age [4,13]. The relatively short duration from publication for most articles (median age four years) and low journal IF (median 2) explain the modest citation numbers (median 6.5) for the selected studies. Nineteen (63%) of the articles used the Arabic-translated tools to assess LBP. This is not surprising as LBP is one of the most common spine pathologies that command the use of PROMs [6,9,49,50]. Most patients (60%) were recruited from physiotherapy and rehabilitation services while most studies (57%) were published in spine, orthopaedic and neurosurgery journals. KSA ranked first in the participant's country (40%) with Egypt second (17%). Previous publications had put Egypt as the Arab country with the highest contribution to spine and neurosurgical research [11,12].

The literature is diverse regarding the CCA assessment of the translated PROMs whether to Arabic [2,3] or other languages [54,55]. The reported ranges of good ratings for the various CCA parameters were: initial translation (33%-65%) [2,3,54,55], synthesis (48%-87%) [2,3,54,55], back translation (37%-53%) [2,3,54,55], expert committee review (7%-62%) [2,3,54,55], pretesting (17%-63%) [2,3,54,55], and submission (0-100%) [2,3,54,55]. The ratings in this review were relatively higher in initial translation (83%), back translation (87%) and expert committee review (83%). However, they were comparable to other reports in the literature in synthesis (77%), pretesting (23%) and submission (47%). This variation is likely to be related to the evaluators' interpretation of the data, the definition of good rating, the discrepancy in the way the data are presented by the various researchers, the clearness of the translators’ qualifications and awareness status about the tool, and the exactness of the role of the various committees (expert committee, developer and central committee).

The literature also varies widely in the appraisal of management properties of translated PROMs whether to Arabic [2,3,10] or other languages [7,54,55]. The reported ranges of good ratings for the various management properties parameters were: internal consistency (20%-94%) [2,3,7,10,54,55], reliability (38%-94%) [2,3,7,10,54,55], structural validity (25%-87%) [2,3,7,10,54,55], cross-cultural validity (3%-38%) [2,3,7,10], floor/ceiling effect (0-83%) [3,7,10,54,55], content validity (0-59%) [2,3,10,54,55], error measurement (0-55%) [2,7,10] and responsiveness (0-44%) [2,3,10,54,55]. The ratings in this review were generally good for internal consistency (87%), reliability (73%), structural validity (63%), and cross-cultural validity (53%). The ratings were relatively lower for floor/ceiling effect (43%), content validity (30%), error measurement (13%) and responsiveness (7%). Apart from a slightly higher rating for cross-cultural validity, the scoring for the parameters is within the wide-ranging results in the literature. The latter is likely to be linked to the reviewers’ understanding of the definitions of the range of parameters particularly when the data are presented in different ways by the various authors.

Limitations

There are several limitations to this study. The study was reliant on the precision of PubMed. It is possible that some Arabic-translated PROMs may have been missed. The exclusion of studies that were not published in English may have caused some bias. The grading of the CCA and management properties of the translated PROMs require a subjective judgement which we strived to reduce by having two of the authors review the studies independently. The guidelines do not provide information on how to assign numeric values to the grading. Also, many of the studies did not provide all the necessary quality-related information. The citation numbers were taken at a certain point which was likely to change relatively quickly. The review did not necessarily provide a specific solution to which Arabic-translated PROMs to be used in specific spine diseases.

## Conclusions

Most of the widely utilized PROMs in spine research, except a few, have Arabic-translated cross-culturally adapted validated versions. The majority of studies were published in recent years and have modest citation numbers. Most participants had low back pain and were recruited from physiotherapy and rehabilitation services. The methodological quality of the studies was good apart from a few shortages that could be improved upon by further research. Work should be done to address the validation of Arabic versions of SF-36, VAS and EQ-5D in spine research. PROMs are valuable in systematizing subjective outcomes. Their usage in research and clinical settings in any validated language should be highly encouraged.
